# Women’s perspectives of female genital cutting: Q-methodology

**DOI:** 10.1186/1472-6874-14-11

**Published:** 2014-01-17

**Authors:** Nazar P Shabila, Abubakir M Saleh, Rojan K Jawad

**Affiliations:** 1 Department of Community Medicine, College of Medicine, Hawler Medical University, Erbil, Iraq; 2 Department of Obstetrics and Gynecology, College of Medicine, Hawler Medical University, Erbil, Iraq

## Abstract

**Background:**

Understanding women’s perspectives of female genital cutting is particularly critical for understanding the roots of the problem and enhancing effectiveness of any prevention program. Very limited research has examined how people in Iraqi Kurdistan Region think about this practice. This study aimed to explore the perspectives of women of female genital cutting with the aim of uncovering discrepancies and commonalities between women of different socio-educational groups.

**Methods:**

An explorative study using Q-methodology was conducted with 29 women from different educational and socio-economic statuses in Erbil, the main city of the Iraqi Kurdistan Region. Participants were asked to rank-order a set of 39 statements about different aspects of female genital cutting into a distribution on a scale of nine from “disagree most” to “agree most”. By-person factor analysis was performed with factors or latent viewpoints extracted through centroid method and varimax rotation.

**Results:**

A four-factor solution and one consensus perspective provided the best conceptual fit for the women’s perspectives about female genital cutting. Factor 1, entitled “positive cultural tradition”, centers on recognizing female genital cutting as a positive cultural aspect and an essential part of the Kurdish culture. Factor 2, “active opponents”, positions around actively opposing the practice of female genital cutting and considering the practice a violation of human rights. Factor 3, “role of law”, stresses the importance of developing and enforcing law for combating female genital cutting. Factor 4, “health concerns and passive opposition”, represents the perspectives of recognizing the importance of health concerns resulting from female genital cutting and opposition of the practice but not in an active manner. A consensus perspective, “marital role”, centers primarily on lack of effect of female genital cutting on women’s marital role.

**Conclusions:**

Female genital cutting is still a contentious issue among women in Iraqi Kurdistan Region. By identifying disagreement and consensus among women, four different perspectives on female genital cutting were uncovered with having perspectives at both extremes of accepting the practice and actively opposing it. The study highlighted the typical characterizations that are associated with each perspective.

## Background

Female genital cutting (FGC), also known as female circumcision or female genital mutilation, is associated with a series of health risks and consequences. It often causes pain and bleeding as immediate consequences of the procedure. Other associated immediate complications include difficulty in passing urine and infection, while long term health risks include chronic pain, chronic infections, poor quality of sexual life, birth complications and psychological consequences [1-3]. FGC is also a clear violation of human rights of girls and women, which could be considered one of the main manifestations of gender inequality and discrimination [4].

FGC is a deeply rooted tradition in more than 28 African countries and a few populations in Asia and the Middle East [5]. It is estimated that 100–140 million women have experienced some form of the practice all over the world [6]. It is also estimated that around 3 million girls in sub-Saharan Africa, Egypt and Sudan, the majority of which below 15 years, are at risk of FGC annually [5].

FGC is widely practiced in Iraqi Kurdistan Region, which is inhabited mostly by Muslim Kurds. According to activists and human rights organizations, the prevalence of FGC in Iraqi Kurdistan Region is around 40% [7]. A recent study from the region have reported a prevalence of 58.6% among women at reproductive age (15-49% years) in Erbil City [8], while another study reported a lower prevalence among the females below 20 year old (23%) [9]. The roots of the practice in Kurdistan Region are unclear. Although the practice is common in Iraqi and Iranian Kurdish areas [10], it is less common in other parts of Iraq and in Kurdish areas in neighboring Turkey. The prevalence of FGC is particularly high in the rural areas of Iraqi Kurdistan Region. In some specific rural areas a prevalence of up to 70% has been reported. Traditionally, Kurdish society is agrarian; a significant part of the population lives outside cities, where the high prevalence of illiteracy and poverty and presence of conservative Islam appear to play a role in the high prevalence of FGC [7,11,12].

Understanding women’s perspectives of female genital cutting is particularly critical for understanding the roots of the problem and enhancing effectiveness of its preventions. While few studies have assessed the prevalence of FGC and its associated factors in Iraqi Kurdistan Region, very limited research has examined how people think about this practice. This study intended to explore the perspectives of women of female genital cutting with the aim of uncovering discrepancies and commonalities between women of different socio-educational groups.

## Methods

Q-methodology is a research method that combines qualitative and quantitative methods and provides a scientific foundation for systematic study of subjectivity and preference through characterizing shared viewpoints among groups of people [13,14]. Typically, in a Q-study a sample of statements about some topic, called the Q-set, are presented to respondents, called the P-set, to rank-order them from their individual point of view using a quasi-normal distribution. Then individual rankings (or viewpoints) are subjected to factor analysis [13,15].

### Development of the Q set

To determine the issues and viewpoints concerning FGC in Iraqi Kurdistan Region a comprehensive review of literature [1,7,8,16-19] and media reports was conducted. As a result of the statement identification step, 172 statements related to FGC were extracted. All the statements were reviewed for similarities and differences. Statements that were repeated were discarded, some statements of close similarity were merged and views which were polar opposite were deleted. Two members of the research team made independent decisions about these statements. The aim was to include statements from various aspects of the problem like the perceived positive and negative effects, religious beliefs, cultural traditions and uncertainties around it. The two researchers compared their results and discussed statements which lacked agreement until consensus was reached. Finally, 39 statements that potentially described and sufficiently represented the problem of FGC in Iraqi Kurdistan Region were selected.

Once the set of statements was confirmed, they were translated to Kurdish language. The translation was validated by a native Kurdish speaker fluent in English language, who translated the Kurdish version of the statements back to English to ensure accuracy. The final set of 39 statements in Kurdish language were numbered randomly and typed onto small cards with one statement per card. After the Q-sample was created, the Q-sort was developed, which involved creating a quasi-normal distribution with a specific number of cells equal to the number of the Q-sample statements. This constituted the data collection instrument for the study.

### The P-set

This study was conducted in Erbil, the main city of Iraqi Kurdistan Region. Selection of study participants was guided by the aim to maximize the possibility that a variety of perspectives could be expressed [20]. The aim was to recruit women who were potentially representative of different socio-economic levels and those who could provide the best insights on this topic. Therefore, the sample was purposively selected to include both single and married women representing different age groups and different educational and socio-economic statuses. As Q-Methodology is a kind of exploratory factor analysis that is not designed for hypothesis testing, it is not typically subjected to sample size calculation. The number of participants is usually, but not necessarily, smaller than the Q set [21]. The aim is to have four or five persons defining each anticipated viewpoint, which are often two to four, and rarely more than six [22]. Therefore a sample size of 29 women was selected.

### Q-sorting

The selected women were invited to participate in the study. Through a one-to-one session the purpose of the study and clear step by step instructions for completing the task were explained to each participant by a female doctor and participant’s consent was obtained. Each participant was asked to sort the cards into 9 piles from -4 (most disagree) to +4 (most agree), in relation to her perception about different aspects of FGC and according to the Q-sort table. For illiterate study participants, a trained female doctor assisted in data collection using a step by step guideline. Through a one-to-one session the female doctor read and explained each statement and asked the participant to distribute the statements into three initial piles of generally agree, generally disagree or neutral/not sure. Then the cards in each pile were revisited with the participant to distribute the cards according to the Q-sort table as per to the participant’s level of agreement or disagreement with the statements. The study was approved by the Ethics Committee of Hawler Medical University.

### Q analysis and factor interpretation

The PQ Method 2.11 program was used for the analysis of Q-sorts [23]. The prominent common viewpoints, known as factors, were extracted using centroid factor extraction and varimax rotation. Factors representing at least two defining sorts and having eigenvalues greater than one were extracted [24]. A conservative significance level of p < 0.01 was chosen for factor loading. Thus, those Q-sorts that achieved a factor loading of 0.413 or above on a given factor were considered to have loaded significantly onto that factor [22]. An explanation of how this is calculated is shown Additional file 1. Several different factor solutions were examined for obtaining the most meaningful, consistent and coherent factors.

The resultant factors represent sorts that were made by individuals who have responded in essentially the same way. Each factor or viewpoint was interpreted subjectively by examining the characterizing (those with a rank value of ‘+4’ , ‘+3’ , ‘-3’ , ‘-4’) and the distinguishing (whose score on that factor is significantly different from its score on any other factor) statements [13]. Distinguishing statements that are significant at p < 0.05 are highlighted with asterisk (*), and those at p < 0.01 are highlighted with double asterisk (**) in the results section. Finally a conceptual interpretation was developed to capture the essence of the viewpoints being endorsed.

## Results

Twenty nine women participated in the study. Their mean ± SD age was 35.6 ± 10.0 years. Details of the participants’ socio-demographic characteristics are shown in Table 1.

**Table 1:** Socio-demographic characteristics of the participants

**Characteristic**	**No.**	**(%)**
**Age (years)**		
16-25	8	27.6
26-35	12	41.4
>35	9	31.0
**Marital status**		
Single	8	27.6
Married	21	72.4
**Employment status**		
Government employee	15	51.7
House wife	5	17.2
Student	4	13.8
Not employed	5	17.2
**Education level**		
Illiterate	3	10.3
Primary school	6	20.7
Secondary school	6	20.7
Institute*	9	31.0
College**	5	17.2
**Mother’s education**		
Illiterate	22	75.9
Educated	7	24.1
**Father’s education**		
Illiterate	15	51.7
Educated	14	48.3
**Place of birth**		
Urban	25	86.2
Rural	4	13.8
**FGC status**		
Yes	15	51.7
No	13	44.8
Don’t know	1	3.4

*Institute; 2 years study after secondary school.

**College; 4 years study after secondary school.

**Figure 1:**
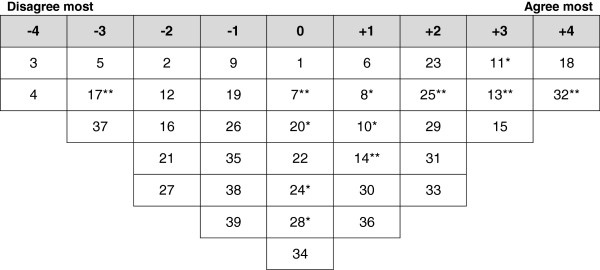
**Ideal Q grid for Factor 1 – positive cultural tradition.** *Distinguishing statement significant at <0.05. **Distinguishing statement significant at <0.01.

**Figure 2:**
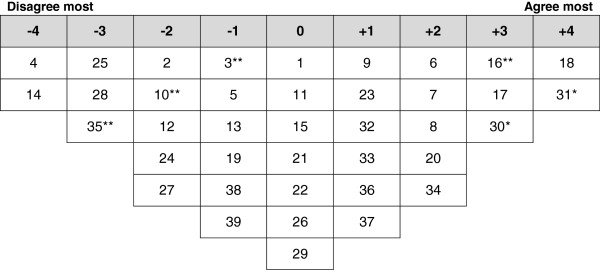
**Ideal Q grid for Factor 2 – active opponents.** *Distinguishing statement significant at <0.05. **Distinguishing statement significant at <0.01.

**Figure 3:**
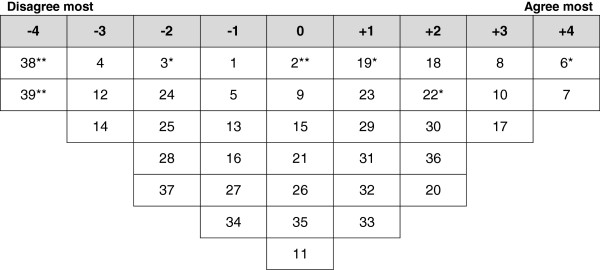
**Ideal Q grid for Factor 3 – role of law.** *Distinguishing statement significant at <0.05. **Distinguishing statement significant at <0.01.

**Figure 4:**
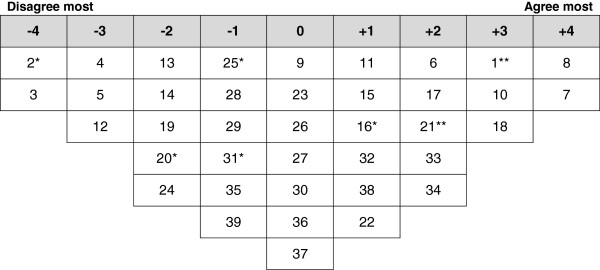
**Ideal Q grid for Factor 4 – health concerns and passive opposition.** *Distinguishing statement significant at <0.05. **Distinguishing statement significant at <0.01.

Analysis of the participants’ Q-sorts resulted in four discrete perspectives (a four factor solution), accounting for 71% of the variance in the correlation matrix (Table 2). One factor reflected positive perspectives of FGC and three factors highlighted negative perspectives. Ideal Q grids have been generated for each of these factors to clearly illustrate the pattern of response characteristics of each factor (Figures 1, 2, 3 and 4).

**Table 2:** Statements and factor scores

**#**	**Statement**	**Factor**			
		**1**	**2**	**3**	**4**
1	There are necessary laws against FGC in Kurdistan	0	0	-1	3**
2	Women with FGC are less likely to catch sexually transmitted infections	-2	-2	0**	-4*
3	FGC improves fertility	-4	-1**	-2*	-4
4*	**One is not a proper woman until she undergo FGC**	**-4**	**-4**	**-3**	**-3**
5	The removal of the clitoris promotes cleanliness	-3	-1	-1	-3
6	People who subject their daughters to FGC should be prosecuted	1	2	4*	2
7	I am not in favor of FGC and think this practice should stop	0**	2	4	4
8	I haven’t seen any religious book that prescribes FGC	1*	2	3	4
9	Males prefer females who have undergone FGC	-1	1	0	0
10	The decision to get girls to undergo FGC is usually taken by grandmother	1*	-2**	3	3
11	I think FGC will soon be history among Kurdish	3*	0	0	1
12*	**Without FGC a woman is unable to fulfill her intended role in marriage**	**-2**	**-2**	**-3**	**-3**
13	If someone in Kurdistan doesn’t perform FGC to their daughter, it may become an embarrassment to the entire family	3**	-1	-1	-2
14	Women being cut is prestigious in Kurdistan	1**	-4	-3	-2
15	The majority of Kurds don’t support FGC, but the surrounding environment is pushing them to subject their daughters to FGC	3	0	0	1
16	In Kurdistan, the majority of girls have not undergone FGC	-2	3**	-1	1*
17	FGC practitioners should be prosecuted	-3**	3	3	2
18**	**I think that the majority of the people I know are against FGC**	**4**	**4**	**2**	**3**
19	Many people in Kurdistan do not subject their daughters to FGC only because they are scared of the law	-1	-1	1*	-2
20	FGC should be voluntary and each family should decide if they want to perform FGC or not	0*	2	2	-2*
21	The girls without FGC are healthier	-2	0	0	2**
22	Sometimes, health provider perform FGC for the sake of doing well for the woman	0	0	2**	1
23**	**FGC is a form of discrimination against girls and women**	**2**	**1**	**1**	**0**
24	FGC ensures a girl’s virginity	0*	-2	-2	-2
25	FGC is a positive cultural aspect and is an essential part of our culture	2**	-3	-2	-1*
26**	**FGC makes a girl more beautiful**	**-1**	**0**	**0**	**0**
27*	**FGC prevents promiscuity in girls**	**-2**	**-2**	**-1**	**0**
28	FGC is a religious obligation	0*	-3	-2	-1
29	FGC in its mild form (cutting only the clitoris) does not lead to any complications; it is therefore acceptable	2	0	1	-1
30	FGC is a violation of human rights	1	3*	2	0
31	I would object if family members intended to subject their daughters to FGC	2	4*	1	-1*
32	FGC can cause psychosocial complications	4**	1	1	1
33**	**For physicians or nurses, performing FGC violates their professional ethics**	**2**	**1**	**1**	**2**
34	FGC can cause long-term complications	0	2	-1	2
35	If the clitoris is not removed, a woman cannot please a man	-1	-3**	0	-1
36**	**FGC can lead to serious complications**	**1**	**1**	**2**	**0**
37	FGC is usually carried out by health professionals	-3	1	-2	0
38	Father has an important role on deciding to perform FGC on the girls	-1	-1	-4**	1
39	Woman without FGC does not have clean hands	-1	-1	-4**	-1

*Distinguishing statement significant at <0.05.

**Distinguishing statement significant at <0.01.

Bold type indicates consensus statement.

The four factors were defined by 20 women (69.0%), whereas two participants did not have a statistically significant load on any of the factors and seven participants were confounded, i.e. loaded significantly on more than one factor. The socio-demographic characteristics and factor loading for each participant on each of the four factors are shown in Additional file 1.

### Factor 1 - positive cultural tradition

Factor 1 accounted for 9% of total variance with the Q-sorts of three participants defining this factor. Of these factor exemplars, all had undergone FGC; one was illiterate, one had primary education and one secondary education. Figure 1 illustrates the ideal grid for this factor.

The factor 1 perspective focused upon considering FGC a positive cultural tradition. The shared viewpoint amongst these defining participants is that FGC is a positive cultural aspect and an essential part of the Kurdish culture (25: 2**) and if someone in Kurdistan doesn’t perform FGC to their daughter, it may become an embarrassment to the entire family (13: 3**). They also considered having undergone FGC is prestigious in Kurdistan Region (14: 1**). They thought that majority of Kurds don’t support FGC, but the surrounding environment is pushing them to subject their daughters to FGC (15: 3). They disagreed with the viewpoints that FGC is usually carried out by health professionals (37: -3) and that FGC practitioners should be prosecuted (17: -3**).

Comparing to the other groups, this group of women least agreed with the statements that they are not in favor of FGC and that this practice should stop (7: 0**) and that they haven’t seen any religious book that prescribes FGC (8: 1*). They most agreed with importance of FGC in ensuring girl’s virginity (24: 0*) and that FGC is a religious obligation (28: 0*). However, these women did not think that FGC would improve fertility (3: -4) or promotes cleanliness (5: -3). They also thought that FGC can cause psychosocial complications (32: 4**) and that the practice will soon be history among Kurdish (11: 3*).

This factor was unique by having two neutral statements related to having the decision to get girls undergoing FGC is usually taken by grandmother (10: 1*) and that FGC should be voluntary and each family should decide if they want to perform FGC or not (20: 0*).

### Factor 2 - active opponents

Factor 2 accounted for 31% of total variance with the Q-sorts of nine participants defining this factor. Of these factor exemplars, four had undergone FGC; one was illiterate and one had primary education. Figure 2 illustrates the ideal grid for this factor.

The factor 2 viewpoint is oriented around actively opposing the practice of FGC and considering the practice a violation of human rights. Defining participants strongly disagreed that FGC is a positive cultural aspect and an essential part of the Kurdish culture (25: -3), FGC is a religious obligation (28: -3) or undergoing FGC is prestigious in Kurdistan (14: -4). They considered FGC a violation of human rights (30: 3*) and indicated the necessity of prosecuting of FGC practitioners (17: 3). They also strongly and significantly indicated that they will object if family members intended to subject their daughters to FGC (31: 4*). They thought that the majority of girls In Kurdistan have not undergone FGC (16: 3**).

In comparison with the other groups, the defining participants least disagreed with the statement that FGC improves fertility (3: -1**). These women did not consider removal of clitoris would affect woman’s ability to please a man (35: -3**) and disagreed that the decision to perform FGC on girls is usually taken by grandmother (10: -2**).

### Factor 3 - role of law

Factor 3 accounted for 20% of total variance with the Q-sorts of six participants defining this factor. Of these factor exemplars, one had undergone FGC; one had primary education and the others had higher education. Figure 3 illustrates the ideal grid for this factor.

The main view highlighted by factor 3 stresses the importance of setting and enforcing law for combating FGC. These women strongly agreed that they are not in favor of FGC and thought that this practice should stop (7: 4). They haven’t seen any religious book that prescribes FGC (8: 3). They strongly disagreed with the views that undergoing FGC is prestigious in Kurdistan (14: -3) and that woman not having undergone FGC does not have clean hands (-39: 4**).

Comparing to other groups, these women disagreed that there are necessary laws against FGC in Kurdistan (1:-1*). They also thought that many people in Kurdistan do not subject their girls to FGC only because they are scared of the law (19: 1*). They thought that sometimes health provider perform FGC for the sake of doing well for the woman (22: 2**). However, they strongly agreed that FGC practitioners and people who subject their daughters to FGC should be prosecuted (17: 3 and 6: 4*, respectively).

These women emphasized the strong role of grandmother in deciding to perform FGC on girls (10: 3) with poor role of father in this matter (38: -4**). Comparing to other groups, they perceived the women with FGC as less likely to catch sexually transmitted infections (2: 0**). This factor was unique by having one neutral statements related to having FGC improving fertility (3: -2*).

### Factor 4 - health concerns and passive opposition

Factor 4 accounted for 11% of total variance with the Q-sorts of two participants defining this factor. Of these factor exemplars, one had undergone FGC and both were well educated. Figure 4 illustrates the ideal grid for this factor.

Factor 4 reflects the perspectives of recognizing the importance of health concerns resulting from FGC and opposition of the practice but not in an active manner. These women were not in favor of FGC and thought that this practice should stop (7: 4). However, they disagreed with the statement that they would object if family members intended to subject their daughters to FGC (31: -1*). They also disagreed with the statement that FGC should be voluntary and each family should decide if they want to perform FGC or not (20: -2*).

These women strongly agreed that they haven’t seen any religious book that prescribes FGC (8: 4) and they thought that there are necessary laws against FGC in Kurdistan (1: 3**). They thought that the decision to get girls undergoing FGC is usually taken by grandmother (10: 3).

The perspective highlighted by factor 3 stresses also the importance of health consequences of FGC. Defining participants agreed that girls not having undergone FGC are healthier (21: 2**). They strongly disagreed with the claims that women with FGC are less likely to catch sexually transmitted infections (2: -4*), FGC improves fertility (3: -4) or removal of clitoris promotes cleanliness (5: -3).

This factor was unique by having two neutral statements related to having FGC as a positive cultural aspect and an essential part of Kurdish culture (25: -1*) and that the majority of girls in Kurdistan have not undergone FGC (16: 1*).

### Consensus statements – marital role

Consensus was apparent for eight statements that did not distinguish between any pair of factors. This perspective focused primarily on lack of effect of FGC on women’s marital role. Defining participants across factors strongly disagreed that one is not a proper woman until she has undergone FGC (4: -3 to -4)* and that without FGC a woman is unable to fulfill her intended role in marriage (12: -2 to -3)*. Consensus was also found around having the majority of the people they know against FGC (18: 2 to 4)**. Women in different groups were neutral or slightly agreed with the statements that physicians or nurses performing FGC violate their professional ethics (33: 1 to 2)**, FGC is a form of discrimination against girls and women (23: 0 to 2)** and FGC can lead to serious complications (36: 0 to 2)**. Women in different groups were neutral or slightly disagreed with the statements that FGC makes a girl more beautiful (26: -1 to 0)** and FGC prevents promiscuity in girls (27: -2 to 0)*.

## Discussion

This study identified four factors reflecting different perspectives of women around FGC. The first factor reflected the view of accepting FGC as a positive cultural tradition. The other three factors shared the view of opposing the practice but with different levels of opposition and with a focus on three different aspects of FGC.

Women in factor 1 perceived FGC as a positive cultural tradition and an essential part of Kurdish culture. This factor reflects the position of those women who still believe in practicing FGC to avoid cultural embarrassment or stigma and maintain a good position in the society. Social and cultural traditions are important reasons for practicing FGC in many settings, which in some instances surpass dictate of religion as the most common reason [19,25]. This is particularly true for Iraqi Kurdistan Region as two other studies have shown that social and cultural traditions are the main reasons for practicing FGC (40.7% to 46.7%) [8,9]. These two studies have also reported a relatively high proportion of women that supported continuation of FGC practice particularly the mutilated participants (36.6% and 28%). One of these studies suggested that the main provocative factor for continuation of the practice is tradition and customs inherited in the family from mothers to daughters [8].

Compared to the other groups, women loading on this factor pointed out to the religious obligation of FGC and were apparently more hesitant or neutral to state that they have not seen any religious book prescribing FGC. Though no religious scripts prescribe FGC, it is often believed that the practice has religious support. Religious leaders take varying positions with regard to FGC: some promote it, some consider it irrelevant to religion, and others contribute to its elimination [26,27]. Two other studies from Kurdistan Region reported dictate of religion as a very important reason for practicing FGC (50.3% and 38.8%) [8,9].

The position of the women loading on factor 2 was active opposition of FGC with considering it a violation of human rights and openly objecting if family members intended to subject their daughters to FGC. Such position might have been raised as a result of increased awareness of these women about the problem of FGC in the region. Such increased awareness might have resulted from the advocacy of civil society organizations and women rights groups through the campaign of “Stop Female Genital Mutilation in Kurdistan” [28]. However, the importance and effectiveness of such active opposition stand of this group of women is potentially limited by their opinion of not recognizing FGC as critical problem in the region as they indicated that the majority of girls in Kurdistan have not undergone FGC. This might also limit their potential active role in combating the FGC problem in the society.

Women loading on factor 3 primarily emphasized the role of law in FGC problem in Kurdistan Region. While they thought that many people do not subject girls to FGC only because they are scared of the law, they recognized the need for more necessary laws or their enforcement. They particularly emphasized the importance of prosecuting FGC practitioners and people who subject their daughters to FGC. Laws prohibiting and criminalizing FGC have been introduced in several countries where FGC is practiced including several African countries [29]. Most industrialized countries, including the majority of Western Europe countries, where immigrant communities continue the practice have either employed already existing general criminal law provisions related to abuse or mutilation or introduced specific criminal law provisions prohibiting FGC [5,30]. In Iraqi Kurdistan Region, the Family Violence Bill that was passed in June 2011 includes several provisions criminalizing the FGC in Kurdistan. The bill listed FGC among13 items of family violence. According to this bill the penalty of encouraging FGC practice is a fine of 1–5 million Iraqi Dinars, while the penalty of FGC performers is a fine of 2–10 million Iraqi Dinars and/or imprisonment for 6 months to 3 years. If the performer is a health professional then the penalty could be more severe and the performer could be banned from practice for a (non-specified) period [31].

Although laws criminalizing FGC are enacted in many settings, their enforcement remains a concern. For example, legislation in UK and Wales has set the penalty for aiding, abetting or counseling to procure FGC to 14 years imprisonment or a fine or both. However, FGC is a hidden practice which is difficult to detect. Therefore, no prosecutions on FGC have been made under the UK legislation [32]. Moreover, the effects of domestic laws on FGC prevalence levels are largely understudied; as an indicator, they need to be more closely monitored [33]. Enforcement of the bill and people’s awareness of its existence and contents is a matter that needs further exploration.

The perspectives around factor 4 were primarily related to recognizing the health concerns resulting from FGC and opposing the practice but not in an active manner. With the recent advocacy and awareness campaigns in the region many people have become more aware of the health concerns related to FGC. However, rejection of a practice that is deeply embedded in the roots of the society cannot be simply achieved by recognizing its harms. Like any other health issue, knowledge alone does not always necessarily affect behavior change particularly in an active manner [34]. Although these people had concerns about the health consequences of FGC, they only referred to the general health issues with no focus of serious complications. This might be related to having the less risky type I FGC as the most common type of FGC practiced in Iraqi Kurdistan Region [8], which involves partial or total removal of the clitoris and/or the prepuce (clitoridectomy) [1]. This might also affect people’s attitude to less actively reject the practice.

Interestingly, the women loading on the different factors had consensus around several aspect of FGC. They had consensus about the notion that FGC will not add to the women’s marital role and maturity. In several societies where FGC is practiced, a girl can't be considered an adult/women until she has FGC and hence a girl cannot marry without going through FGC [35]. However, this notion does not seem to be an important reason for performing FGC in Iraqi Kurdistan Region. The study participants also agreed that the majority of the people they know are against FGC. This is interesting as why FGC is widely practiced while majority of people are against it. This needs more in-depth exploration. It is also striking that women in the different groups were more or less neutral about important and serious notions of FGC including notions related to gender rights, professional ethics, serious health risks and some cultural beliefs of benefits of FGC. Limited concern about FGC complications might be attributed to having high proportion of type I FGC in the region which has few complications. Another study from Erbil City showed that type I FGC accounts for 99.6% of cases and only 6.3% of FGC victims reported they had complications [8].

### Limitations

This study has some potential limitations. It is merely an exploration of the perspectives and a range of viewpoints about FGC that are embedded in women population. The study is not meant to be representative as Q-studies are explorative rather than potential generalizable studies. Even though the viewpoints of the less educated people are usually overlooked in Q-studies as administration of Q-sort requires the respondent to have a certain level of education, we decided to involve the uneducated and less educated women in the study. This required additional time and efforts from both the participants and the data collection facilitator to administer the questionnaire. Direct supervision by the female doctor on the data collection process might have jeopardized women’s actual opinions as sensitive health problems can be under-reported in face-to-face interviews compared with self-administered questionnaires [36]. It is expected that some women in the society deny acceptance of FGC particularly in societies where legislation against FGC is well established and enforced. However, some other women might claim that they accept FGC because social pressure is high and stating that they are against FGC risks them to be ostracized. Therefore, expression of subjective opinion of this group of illiterate women under direct supervision of data collector might have introduced bias to the study results in either direction of accepting or opposing FGC practice.

## Conclusions

FGC is still a contentious issue among women in Iraqi Kurdistan Region. By identifying disagreement and consensus among women, four different perspectives on FGC were uncovered with having perspectives at both extremes of accepting the practice and actively opposing it. The typical characterizations that are associated with each perspective were highlighted. This study has demonstrated the complexity of perspectives about FGC, which has practical implications for those working on fighting this practice.

## Abbreviations

FGC: Female genital cutting.

## Competing interests

The authors declare that they have no competing interests.

## Authors’ contributions

NPS, RKJ and AMS conceptualized and designed the study. RKJ collected the data. NPS and AMS carried out data analysis and interpretation. NPS prepared the manuscript. RKJ and AMS extensively reviewed and edited the manuscript. All authors read and approved the final manuscript.

## Pre-publication history

The pre-publication history for this paper can be accessed here:

http://www.biomedcentral.com/1472-6874/14/11/prepub

## Supplementary Material

Additional file 1 Participants’ characteristics and factor loading on the four factors. Click here for file

## References

[B1] OHCHR, UNAIDS, UNDP, UNECA, UNESCO, UNFPA, UNHCR, UNICEF, UNIFEM, WHO Eliminating female genital mutilation: An interagency statement 2008 Geneva: WHO

[B2] UNICEF Changing a harmful social convention, female genital mutilation/cutting 2005 Italy: Innocenti digest

[B3] WHO A systematic review of the health complications of female genital mutilation including sequel in childbirth 2000 Geneva: WHO

[B4] UNICEF Female genital mutilation/cutting: a statistical exploration 2005 New York: UNICEF

[B5] WHO Female genital mutilation-new knowledge spurs optimism Prog Sex Reprod Health Res 2006 72 1

[B6] WHO Female genital mutilation, integrating the prevention and the management of the health complications into the curricula of nursing and midwifery: A teacher’s guide 2001 WHO: Geneva

[B7] WADI Female genital mutilation in Iraqi-Kurdistan: An empirical study by WADI 2010 Frankfurt: WADI

[B8] Yasin BA Al-Tawil NG Shabila NP Al-Hadithi TS Female genital mutilation among Iraqi Kurdish women: a cross-sectional study from Erbil City BMC Public Health 2013 13 1 809 10.1186/1471-2458-13-809 24010850 PMC3844478

[B9] Saleem RA Othman N Fattah FH Hazim L Adnan B Female genital mutilation in Iraqi Kurdistan: description and associated factors Women Health 2013 53 6 537 551 10.1080/03630242.2013.815681 23937728

[B10] Pashaei T Rahimi A Ardalan A Felah A Majlessi F Related factors of female genital mutilation (FGM) in Ravansar (Iran) J Womens Health Care 2012 1 108

[B11] Markey P Fighting female genital mutilation (FGM) in Iraqi Kurdistan, one Kurdish village at a time http://www.ekurd.net/mismas/articles/misc2012/10/state6569.htm

[B12] von der Osten-Sacken T Uwer T **Is female genital mutilation an Islamic problem**? Middle East Q 2007 14 1 29 36

[B13] van Exel NJA de Graaf G Q methodology: A sneak preview 2005 http://qmethod.org/articles/vanExel.pdf

[B14] Shinebourne P Adams M Q-methodology as a phenomenological research method Existent Anal 2007 18 1 103 116

[B15] Smith NW Current systems in psychology: history, theory, research, and applications 2001 Belmont, CA: Wadsworth

[B16] Avén J Jacobson CA Nursing students’ knowledge of and attitudes towards female genital mutilation: a quantitative study in Ghana Bachelors thesis 2011 Stockholm: Red Cross University College of Nursing

[B17] Gele AA Kumar B Hjelde KH Sundby J Attitudes toward female circumcision among Somali immigrants in Oslo: a qualitative study Int J Womens Health 2012 4 7 17 22312195 10.2147/IJWH.S27577 PMC3271810

[B18] Onuh SO Igberase GO Umeora JOU Okogbenin SA Otoide VO Gharoro EP Female genital mutilation: knowledge, attitude and practice among nurses J Natl Med Assoc 2006 98 3 409 414 16573307 PMC2576104

[B19] Okeke TC Anyaehie USB Ezenyeaku CCK An overview of female genital mutilation in Nigeria Ann Med Health Sci Res 2012 2 1 70 73 10.4103/2141-9248.96942 23209995 PMC3507121

[B20] Cross RM Exploring attitudes: the case for Q methodology Health Educ Res 2005 20 2 206 213 15385430 10.1093/her/cyg121

[B21] Brouwer M Q is accounting for tastes J Advertising Res 1999 39 2 35 39

[B22] Stainton Rogers R Smith JA, Harre R, Van Langenhove L Q methodology Rethinking methods in psychology 1995 Thousand Oaks, CA: Sage 178 192

[B23] Schmolck P PQMethod software 2002 http://schmolck.userweb.mwn.de/qmethod/

[B24] Shinebourne P Using Q method in qualitative research IJQM 2009 8 1 93 97

[B25] Tag-Eldin M Gadalla M Altayeb N Prevalence of female genital mutilation among Egyptian girls Bull World Health Org 2008 86 241 320 10.2471/BLT.07.042093 PMC2647417 18438515

[B26] WHO Female genital mutilation fact sheet 2013 http://www.who.int/mediacentre/factsheets/fs241/en/

[B27] Al-Zalmi MI Female genital mutilation: side effects and its banning in Quran 2011 Erbil: Shahab Press Arabic

[B28] Stop Violence Against Women New law criminalizing female circumcision in Iraq http://www.stopvaw.org/New_Law_Criminalizing_Female_Genital_Mutilation_in_Iraq.html

[B29] Center for Reproductive Rights Female genital mutilation (FGM): Legal prohibitions worldwide 2008 New York: Center for Reproductive Rights

[B30] Poldermans S Combating female genital mutilation in Europe: A comparative analysis of legislative and preventative tools in the Netherlands, France, the United Kingdom, and Austria MSc thesis 2006 Vienna: University of Vienna

[B31] Iraqi Kurdistan Parliament Law number 8: Combating family violence in Iraqi Kurdistan Region Waqaehi Kurdistan 2011 122 6 9 Arabic

[B32] Dorkenoo E Morison L Macfarlane A A statistical study to estimate the prevalence of female genital mutilation in England and Wales 2007 UK: Foundation for Women’s Health, Research and Development (FORWARD)

[B33] UNICEF Female genital mutilation/cutting: A statistical overview and exploration of the dynamics of change 2013 New York: UNICEF

[B34] Scott SD Albrecht L O'Leary K Ball GD Hartling L Hofmeyer A Jones CA Klassen TP Kovacs Burns K Newton AS Thompson D Dryden DM Systematic review of knowledge translation strategies in the allied health professions Implement Sci 2012 7 70 10.1186/1748-5908-7-70 22831550 PMC3780719

[B35] Dalal K Lawoko S Jansson B Women’s attitudes towards discontinuation of female genital mutilation in Egypt J Inj Violence Res 2010 2 1 41 47 10.5249/jivr.v2i1.33 21483197 PMC3134892

[B36] Bowling A Mode of questionnaire administration can have serious effects on data quality J Public Health 2005 27 3 281 291 10.1093/pubmed/fdi031 15870099

