# Supplementary material for: Women’s perspectives of female genital cutting: Q-methodology
Source: BMC Womens Health. 2014 Jan 17;14:11. doi: 10.1186/1472-6874-14-11 (PMC3898223; doi:10.1186/1472-6874-14-11)
Supplement: Additional file 1 — Participants’ characteristics and factor loading on the four factors. [file 1472-6874-14-11-S1.pdf]

Additional files

Additional file 1 – Participants' characteristics and factor loading on the four factors

Participant No.	Education level*	FGC status	Factor loading			
			1	2	3	4
1	Primary	Yes	0.141	0.294	**0.822**[X]	0.234
2	Institute	No	0.085	**0.874**[X]	0.208	0.319
3	Institute	Yes	0.001	**0.860**[X]	0.327	0.233
4	Illiterate	No	0.024	**0.923**[X]	0.239	0.126
5	College	Yes	0.147	**0.864**[X]	0.287	0.254
6	Secondary	No	0.066	**0.811**[X]	0.363	0.169
7	Institute	No	0.104	**0.867**[X]	0.317	0.215
8	Primary	Yes	-0 050	**0.791**[X]	0.286	0.253
9	Illiterate	Yes	**0.522**[X]	-0.104	-0.003	-0.088
10	Institute	No	0.388	0.328	**0.430**[X]	0.321
11	Primary	Don't know	0.280	-0.036	0.108	0.080
12	Illiterate	Yes	0.343	0.340	0.155	0.154
13	Secondary	Yes	0.252	0.312	**0.451**	**0.458**
14	College	No	0.151	**0.469**	0.334	**0.490**
15	Institute	Yes	0.393	**0.487**	**0.461**	0.259
16	College	No	0.378	**0.614**[X]	0.407	0.312
17	Primary	Yes	0.107	**0.483**	0.338	**0.513**
18	College	Yes	0.108	**0.879**[X]	0.150	0.264
19	Institute	Yes	0.146	0.233	0.194	**0.753**[X]
20	College	No	0.297	0.353	0.392	**0.661**[X]
21	Secondary	Yes	**0.502**[X]	0.358	0.140	0.366
22	Institute	No	**0.423**	**0.493**	0.290	0.352
23	Primary	Yes	**0.694**[X]	0.111	0.162	0.143
24	Primary	Yes	0.184	**-0.468**	**-0.426**	**-0.518**
25	Institute	No	0.196	0.288	**0.852**[X]	0.188
26	Institute	No	0.176	0.266	**0.848**[X]	0.216
27	Secondary	No	0.245	0.371	**0.812**[X]	0.220
28	Secondary	No	0.237	0.358	**0.838**[X]	0.219
29	Secondary	Yes	**0.520**	**0.567**	0.275	0.034
Eigenvalues			1.77	15.77	1.80	1.14
Defining sorts			3	9	6	2
Explained variance %			9	31	20	11
Explained variance cumulative %			9	40	60	71

FGC: Female genital cutting

* Institute; 2 years study after secondary school, College; 4 years study after secondary school.

Bold type indicates significant loadings. Significance at the 1% level is taken as a factor loading greater than (2.58 x 1√n), where n= the number of statements - so in this case significant loadings are those higher than 0.413.

[X] indicates defining sorts.
